# Comprehensive detection of germline variants by MSK-IMPACT, a clinical diagnostic platform for solid tumor molecular oncology and concurrent cancer predisposition testing

**DOI:** 10.1186/s12920-017-0271-4

**Published:** 2017-05-19

**Authors:** Donavan T. Cheng, Meera Prasad, Yvonne Chekaluk, Ryma Benayed, Justyna Sadowska, Ahmet Zehir, Aijazuddin Syed, Yan Elsa Wang, Joshua Somar, Yirong Li, Zarina Yelskaya, Donna Wong, Mark E. Robson, Kenneth Offit, Michael F. Berger, Khedoudja Nafa, Marc Ladanyi, Liying Zhang

**Affiliations:** 1 Department of Pathology, Memorial Sloan Kettering Cancer Center, 1275 York Ave, Box 36, New York, NY 10065 USA; 2 Human Oncology and Pathogenesis Program, Memorial Sloan Kettering Cancer Center, New York, NY USA; 3 Department of Medicine, Memorial Sloan Kettering Cancer Center, New York, NY USA; 4 Illumina Inc, Santa Clara, CA USA; 5 Brigham and Women’s Hospital, Boston, MA USA

## Abstract

**Background:**

The growing number of Next Generation Sequencing (NGS) tests is transforming the routine clinical diagnosis of hereditary cancers. Identifying whether a cancer is the result of an underlying disease-causing mutation in a cancer predisposition gene is not only diagnostic for a cancer predisposition syndrome, but also has significant clinical implications in the clinical management of patients and their families.

**Methods:**

Here, we evaluated the performance of MSK-IMPACT (Memorial Sloan Kettering-Integrated Mutation Profiling of Actionable Cancer Targets) in detecting genetic alterations in 76 genes implicated in cancer predisposition syndromes. Output from hybridization-based capture was sequenced on an Illumina HiSeq 2500. A custom analysis pipeline was used to detect single nucleotide variants (SNVs), small insertions/deletions (indels) and copy number variants (CNVs).

**Results:**

MSK-IMPACT detected all germline variants in a set of 233 unique patient DNA samples, previously confirmed by previous single gene testing. Reproducibility of variant calls was demonstrated using inter- and intra- run replicates. Moreover, in 16 samples, we identified additional pathogenic mutations other than those previously identified through a traditional gene-by-gene approach, including founder mutations in *BRCA1*, *BRCA2*, *CHEK2* and *APC*, and truncating mutations in *TP53, TSC2, ATM* and *VHL*.

**Conclusions:**

This study highlights the importance of the NGS-based gene panel testing approach in comprehensively identifying germline variants contributing to cancer predisposition and simultaneous detection of somatic and germline alterations.

**Electronic supplementary material:**

The online version of this article (doi:10.1186/s12920-017-0271-4) contains supplementary material, which is available to authorized users.

## Background

Our understanding of the genetic basis of cancer susceptibility has improved significantly in the last 30 years. Highly penetrant cancer predisposition genes (defined as genes in which germline mutations confer increased risks of cancer) were identified and shown to cause hereditary cancer syndrome with Mendelian modes of inheritance. To date, more than 100 of such genes have been identified, providing important scientific insights in the molecular mechanisms of cancer initiation, development and progression. Identifying whether a cancer is the result of an underlying disease-causing mutation in a cancer predisposition gene is not only diagnostic for a cancer predisposition syndrome, but also has significant clinical implications in the clinical management of patients and their families. In turn, this has potential to provide substantial cost-effective health benefits with respect to cancer treatment of patients and cancer prevention in healthy individuals [1–3].

The effective management of patients with clinical presentations of cancer predisposition syndromes relies on the accurate, comprehensive and high-throughput clinical assays to identify all disease-causing mutations present in the patients. Massively parallel next-generation sequencing (NGS) technology fulfills this need because it enables the unbiased identification of mutations across the genome or across more targeted regions with high sensitivity and specificity. In multiplex testing, the simultaneous interrogation of target genes of interest allows for an efficient and cost-effective method of screening panels of cancer genes concurrently [4, 5]. Several academic and commercial labs have established and implemented targeted cancer gene panel testing [1, 6–8].

The MSK-IMPACT (Integrated Mutation Profiling of Actionable Cancer Targets) assay is a comprehensive molecular profiling platform, utilizing solution-phase exon capture and next generation sequencing to detect somatic genetic alterations in FFPE tumor specimens. We designed custom DNA probes corresponding to all exons and selected introns of 341 oncogenes and tumor suppressor genes, including all genes that are “druggable” by approved therapies or are targets of experimental therapies being investigated in clinical trials at our Institute [9].

**Fig. 1:**
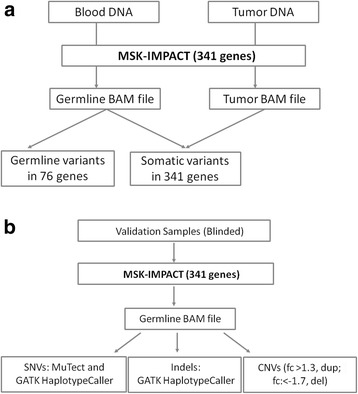
**a**: The MSK-IMPACT workflow. MSK-IMPACT is performed as a matched tumor/normal test at our institution, allowing for concurrent identification of somatic mutations in the tumor sample and inherited germline variants in the subset of 76 cancer relevant genes. **b**: The validation approach. DNA samples that were previously tested positive for a pathogenic or likely pathogenic variant were identified and blinded for the validation. The samples were tested through the MSK-IMPACT pipeline. Three different types of variants (SNVs, indels and CNVs) were called using various analysis tools

Since the MSK-IMPACT panel contains 76 cancer predisposition genes of clinical interest, we sought to validate the detection of germline mutations in these genes and subsequent reporting in a clinical setting. More importantly, as MSK-IMPACT is being performed at our institution as a matched tumor/normal test, we are in a unique position to enable simultaneous detection of germline and unambiguous somatic alterations in a clinical setting (Fig. 1a). Here, we describe the analytic validation and a custom analysis pipeline to detect single nucleotide variants (SNVs), small insertions/deletions (indels <30 bp), copy number variants (CNVs), and structural variants (SVs). We assessed the accuracy and reproducibility of MSK-IMPACT to detect germline variants in normal blood, using a set of 223 samples previously determined to be positive for germline mutations/variants by independent methods. Moreover, since these specimens were initially tested for one or a couple genes related to the clinical symptoms of the diseases, we identified incidental pathogenic mutations in other cancer predisposition genes that would have otherwise been undiscovered by a traditional gene-by-gene approach. This study demonstrated the importance of broad NGS-based, cancer-panel testing in identifying germline genetic mutations contributing to various cancer spectrums in different families.

## Methods

### DNA samples

DNA samples used for validation experiments were derived from 228 unique blood samples. All samples were identified to carry one or more mutations through standard clinical testing in a Clinical Laboratory Improvement Amendments (CLIA) certified laboratory. Clinical testing was performed using a combination of Sanger sequencing, semi-quantitative PCR and MLPA (multiplex ligation-dependent probe amplification). Samples were anonymized and associated metadata were de-identified prior to analysis.

### Sequencing and analysis workflow

We used the same probes for the MSK-IMPACT panel and capture protocol as previously published [9]. The variant calling procedures were modified for germline mutations: SNVs, indels and CNVs (large deletions/duplications). SNV calls are the union of output from two tools: MuTect version 1.1.4 [10] and GATK Haplotype caller (GATK version 2.3.9). GATK HaplotypeCaller (HTC) calls SNVs and Indels simultaneously from read data. Unlike HTC, MuTect [10] does not require correct assembly of a haplotype block prior to identification of SNVs. We determined that the following thresholds on coverage depth (DP) and variant frequency (VF) can detect the expected variants and reject almost all false positive calls: for exonic SNVs and Indels, DP ≥ 50X and VF ≥ 20%; for all other events, DP ≥ 50X and VF ≥ 25%. Large deletions and duplications were identified as germline copy number variant (CNVs), using an in-house algorithm that detects whole gene and partial gene (intragenic) gains or losses The following criteria is used to determine significance of gain or loss events: fold change > 1.3 (single copy gain) or < −1.7 (single copy loss), p < 0.05. DELLY version 0.3.3 [11] was used to detect structural variants in germline samples, using an unmatched reference normal as a control. The structural abnormalities validated here only include large deletions or duplications and do not include translocations or inversions. Figure 1b shows the approaches taken for the validation of the mutation types validated in this study. Additional details can be found under Additional file 1: Supplementary Methods.

### Variant annotation

Annovar [12] is used to annotate calls made from MuTect [10] and GATK HaplotypeCaller [13]. Annovar also checks for the presence of each variant in a number of external databases, such as ClinVar [14], dbSNP [15], the 1000 Genomes cohort data [16] and the NHLBI Exome Sequencing Project cohort [17].

We used the scoring scheme outlined in the ACMG guidelines for variant interpretation [18, 19] to classify SNV and Indel variants as pathogenic (Class 5), likely pathogenic (Class 4), VUS (Class 3), likely benign (Class 2) or benign (Class 1).

## Results

### Target region coverage and statistical determinations of variant calling criteria

The MSK-IMPACT assay is a hybridization capture based assay targeting all exons and selected introns of 341 cancer genes, and has been previously validated for the detection of somatic mutations as a paired tumor/normal sequencing test. At high sequencing depths, a modified workflow using the MSK-IMPACT assay should readily identify germline mutations, since they usually present with high variant frequencies, being either homozygous or heterozygous. Not all 341 genes in the assay are relevant for germline cancer predisposition testing – we chose to focus on a subset of 76 genes with clearly documented associations for various cancer syndromes (the full list of genes, their reference transcripts and corresponding indications of interest can be found in Additional file 1: Table S1). This list contains 26 genes that are also part of the ACMG recommendations for reporting of incidental findings in clinical NGS testing [20].

**Fig. 2:**
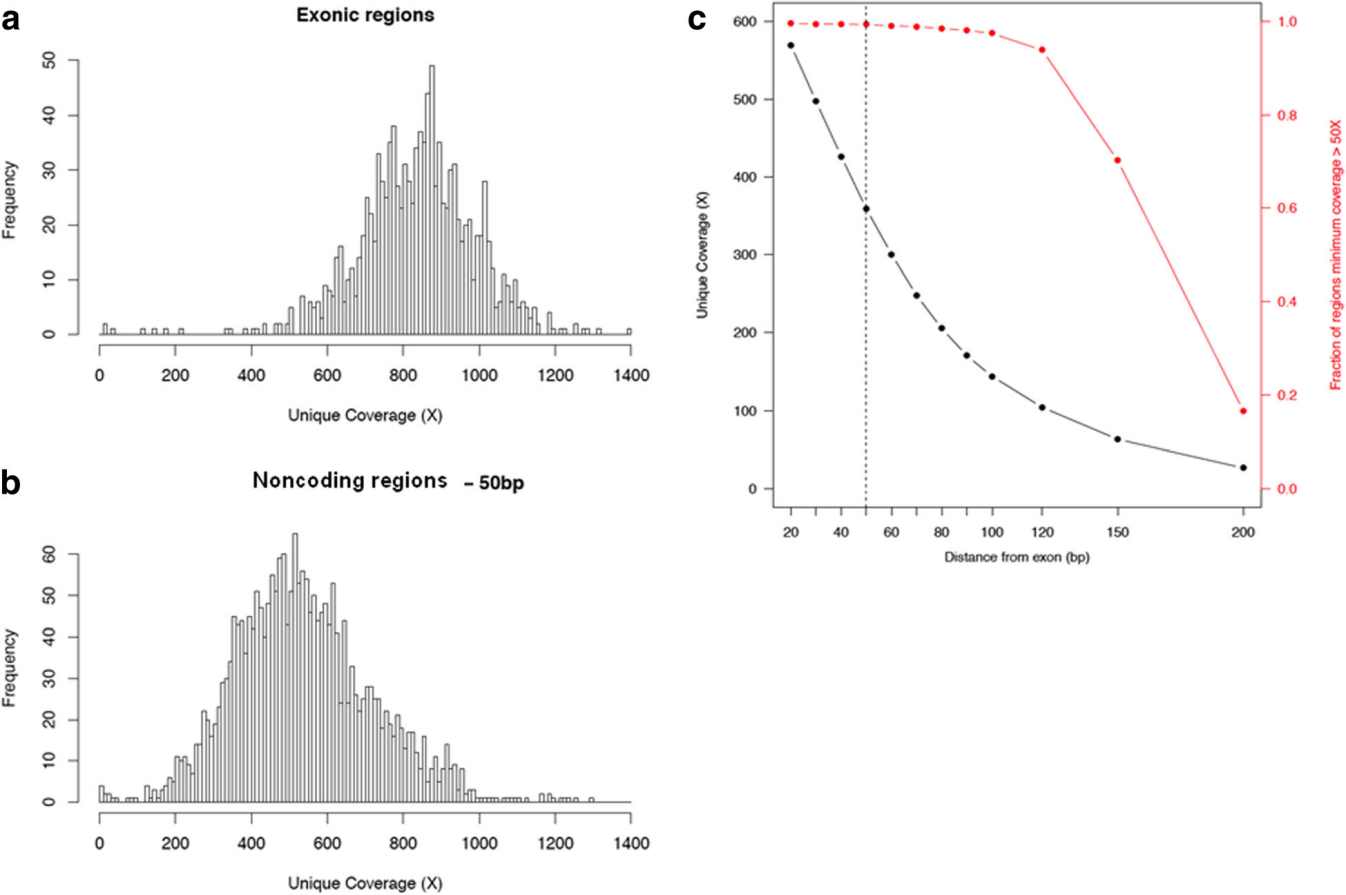
Distribution of sequence coverage. **a** exons of canonical transcripts of 76 cancer predisposition genes within the MSK-IMPACT panel, **b** intronic regions flanking targeted exons (50 bp). **c** Average sequence coverage decreases with increasing distance from the exon-intron boundary (*black line*), while the fraction of intronic regions flanking the exons that maintain a minimum of 50× coverage (*red line*) drops off sharply as the size of the flanking regions exceed 100 bp. *Dotted line* indicates 50 bp

The canonical transcripts for these 76 genes contain a total of 1166 exons and we sequenced a total of 20 blood samples from individuals who had MSK-IMPACT testing, to provide an assessment of the sequencing coverage across these targeted regions. Of note, the MSK-IMPACT capture panel included baits tiling the edges of each exon to ensure high levels of coverage across all exonic positions – as a result, coverage typically extended past the boundaries of an exon into the noncoding sequence. Since these noncoding regions could potentially contain mutations affecting splicing of neighboring exons, we additionally computed levels of sequencing coverage for varying distances into the flanking regions. The average sample coverage across the 20 blood normal samples tested was 787X. Coverage in the exonic regions of the 76 genes of interest was high: the mean coverage across exons was 844X (95% confidence interval: 538X-1116X, Fig. 2a) and all but 8 of the exons were covered to a minimum of 50X (99.3%). It should be noted for the analytical validation of the assay, exons were not assessed based on their average coverage, but on the lowest level of coverage across all nucleotide positions within the exons. Consistently poorly performing exons were excluded from further analysis. Coverage remained high in the flanking intronic regions –up to 50 bp away from the exon boundaries were covered to a minimum of 360X on average – in fact, this distance could be increased as much as 100 bp without significantly affecting the number of noncoding regions with sufficient coverage (defined here as ≥50X, Fig. 2b).

Having demonstrated a high depth of coverage in genomic regions of interest, we sought to quantify limits on sequencing coverage depth and allele frequency needed to avoid potential false positives and negatives. By performing a power analysis, we showed that with 17X coverage, we would be able to detect heterozygous variants (50% allele frequency) with 99% sensitivity (Additional file 1: Table S5). In terms of false positive rejection, we performed a reproducibility analysis on replicates of 13 control samples, where we determined that all non-reproducible variant calls (i.e., noise artifacts) could be filtered out using a coverage depth threshold of 50X and variant frequency threshold of 20% for exonic variants, 25% for variants in noncoding regions (Full analysis details can be found in Additional file 1: Table S6 and Additional file 2: Figure S4 ). Since the coverage of all known pathogenic variants is well above 50X, our assay is well powered for mutation detection.

### Assessment of accuracy in samples with previously confirmed findings

**Fig. 3:**
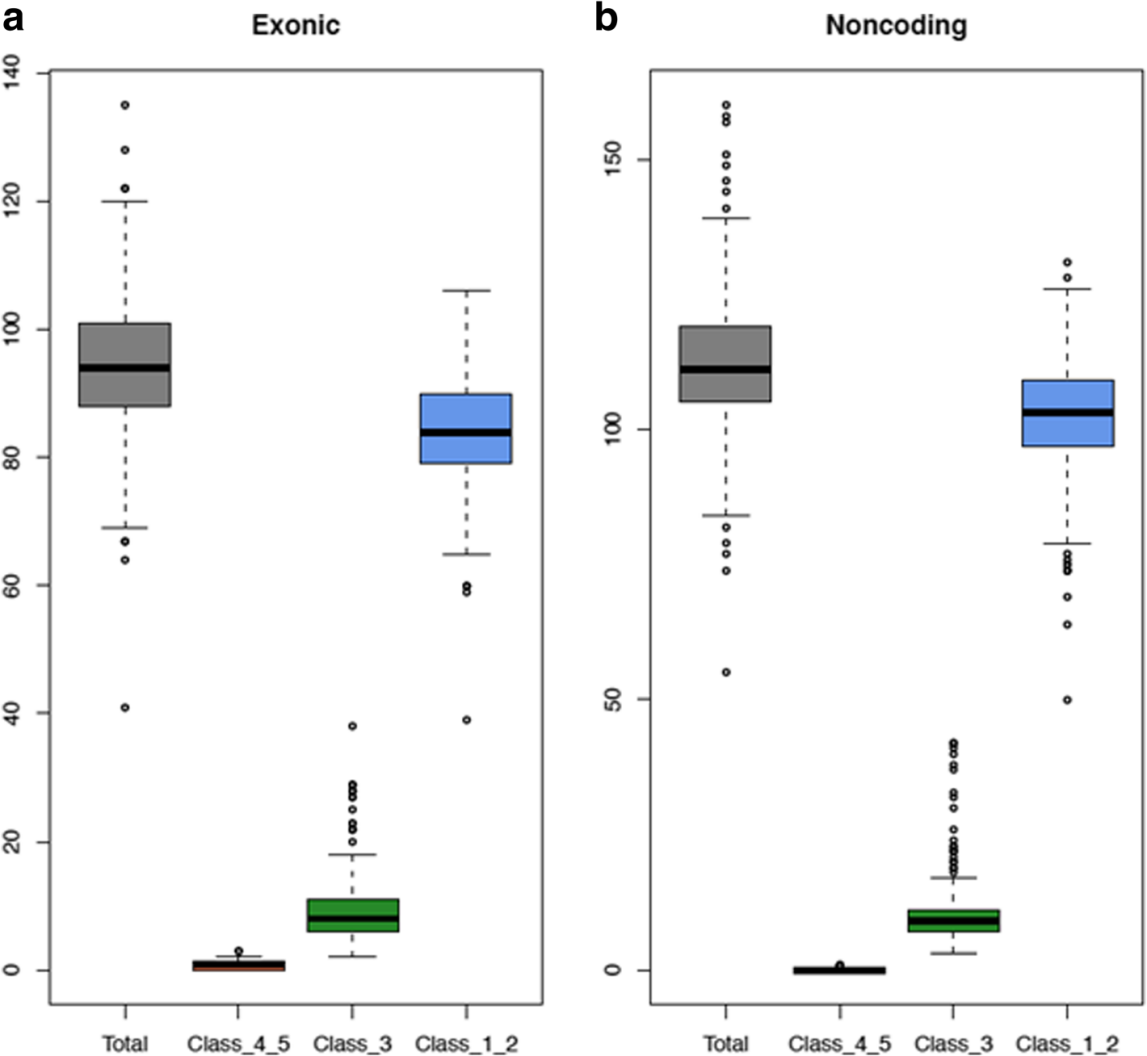
Number of exonic and non-coding mutations identified per sample. **a** Exonic and **b** Non-coding mutations identified per sample tested in the validation study, shown with ranges in a box-and-whisker plot. Distributions are also shown for variants grouped by pathogenicity classification: pathogenic and likely pathogenic = Class_4_5, VUS = Class 3, likely benign and benign = Class_1_2. Pathogenicity classifications are a combination of known pathogenicity determinations for the expected variants, and pathogenicity estimates for incidental variants

All samples used for validation were anonymized prior to analysis. We tested a total of 233 unique samples with previously confirmed variants in 22 cancer predisposition genes: 94 samples were positive for SNVs, 95 samples for indels and 44 samples for germline CNVs (large deletions and duplications), with the majority (168 out of 228, 74%) positive for germline variants in *BRCA1*, *BRCA2*, *MSH2*, *MLH1* and *APC*. (A detailed listing of samples tested per gene can be found in Table 1). These samples had been previously tested in a CLIA-certified laboratory for the presence of a germline mutation using independent technologies: i.e., Sanger sequencing for SNVs and indels, and a combination of semi-quantitative PCR, MLPA, and array CGH for germline CNVs. Most, but not all, of these variants had previously been determined to be pathogenic. On average, each sample reported 94 exonic variants (92 SNVs, 2 indels), of which the majority had greater than 5% frequency in the general population (ExAC, 1000 Genomes, NHLBI ESP), which was sufficient evidence per ACMG guidelines (*BA1)* to consider these variants as benign. There were slightly more variants called in the noncoding regions (112 on average, comprising 103 SNVs and 9 indels) – again, the majority of them were common polymorphisms and were classified as benign/likely benign (Fig. 3).

**Table 1:** List of genes with positive samples tested in the validation study

Gene	Total samples tested	With Known SNVs		With Known Indels		With Known CNVs
		Exons Tested	*N*	Exons Tested	*N*	*N*
APC	14	4,8,16	9	5,16	4	1
ATM	1	-	-	10	1	-
BAP1	1	-	-	8	1	-
BMPR1A	1	-	-	-	-	1
BRCA1	48	2,3,4,6,10,12,13,17,20,23	13	2,3,7,8,10,14,15,16,19,23	23	12
BRCA2	38	3,4,9,11,12,14,18,23,27	12	5,10,11,14,15,17	21	5
CDH1	12	1,3,5,7,10,14	7	8,10,12,15	4	1
CDKN2A	2	2	2	-	-	-
CHEK2	1	11	1	-	-	-
EGFR	1	20	1	-	-	-
EPCAM	1	-	-	-	-	1
FH	5	1,8	2	7,8	2	1
MLH1	26	1,4,7,12,14,19	7	1,2,9,10,11,12,16,19	16	3
MSH2	44	2,3,5,6,7,8,10,12	21	6,7,12,13	6	17
MSH6	10	-	-	4,5,6,8,9,10	10	-
MUTYH	10	2,7,10,12,13,15,16	8	10,14	2	-
PALB2	5	4,7,9	3	-	-	2
PTEN	4	1,5	3	3	1	-
RB1	2	12	1	22	1	-
SMAD4	2	-	-	10,12	2	-
STK11	2	5	1	1	1	-
TP53	3	2,3,10	3	-	-	-
TOTAL	233		94		95	44

5 samples tested were known to be positive for more than 1 germline variant. These mutations included: 1) MSH6 and MLH1 frameshift indels, 2) MSH2 and EPCAM large deletions, 3) MUTYH frameshift indel and p.Y179C missense mutation, 4) MSH2 large deletion and p.P616R missense mutation and 5) APC frameshift indel and p.L1129S

In the subset of 186 unique cases with known SNVs and indels, we successfully detected all 189 known variants (Additional file 1: Table S2A) at high sequencing coverage and at the expected allele frequencies (some samples contained more than 1 known variant, Additional file 3: Figure S1). 96% of SNVs (exonic and noncoding) detected in this validation study had at least 100× coverage, with over 90% of them reported at allele frequencies between 40 and 60% (heterozygous). Sequencing coverage for the indels was a little lower (90% of indels detected had at least 100× coverage) and the allele frequencies for the noncoding indels tended to skew outside the expected ranges for typical heterozygous mutations (only 60% of mutations were reported between 40 and 60% allele frequency).

To assess the ability of the assay to detect large deletions and duplications, we tested a total of 44 unique samples with previously confirmed germline CNVs in 10 genes (Additional file 1: Table S2B) – samples were chosen to include whole gene deletions, as well as challenging cases where only a single exon was deleted or duplicated. To enable automated detection of germline CNVs, we developed an in-house CNV analysis pipeline for assessing significance of whole gene and intragenic copy number variants. Additional file 4: Figure S2 shows examples of germline CNVs called by our algorithm in MSH2 and BRCA2. Our algorithm successfully detected the known germline CNV variant in all 43 tested samples. Of note, we were able to more clearly define breakpoints of the CNV event by incorporating a structural variant (SV) calling algorithm (DELLY) into the pipeline. Additional file 5: Figure S3 shows an example of an intragenic deletion in BRCA1 that was detected by the coverage analysis as a deletion of exons 11 to 18. However, upon review of the SV analysis results, we determined this variant would be more accurately described as a single copy deletion starting in the middle of exon 10 and extending to the 5’ end of exon 18.

### Reproducibility of detected variants

We tested the within-batch (intra-assay) and across-batch (inter-assay) analytic reproducibility of the variant calls using samples with previously confirmed SNVs (*MSH2* p.Ala636Pro), Indels (*BRCA1* and *BRCA2* frameshift indels) and large deletions only (*BRCA1* and *MSH2*) (Additional file 1: Table S3). Experiments were designed to include three replicates of each sample within a single run, and across three different runs. Replicates tested were assigned different barcodes to ensure reproducibility of results regardless of sample/barcode combination. In each case, the known variant was reproducibly detected at similar levels of coverage (raw and normalized) and allele frequency. Furthermore, the total number of variants called (exonic and noncoding) were similar across replicates – in particular, while the number of noncoding variants called in the noncoding regions (50 bp) varied across replicates, the number of exonic variants reported was identical. Upon further inspection, we determined that most of these differences were due to ‘borderline’ variants, i.e., variants that barely pass the filtering criteria of 50X coverage and 25% allele frequency (Additional file 1: Table S4). This is expected in the noncoding regions, since they were not deliberately captured by panel baits (lower coverage than exonic regions, as a consequence) and tend to contain homopolymer repeat regions that affect the local realignment (resulting in lower allele frequencies for detected variants).

### Burden of incidental pathogenic variants in samples with previously confirmed germline mutations

**Fig. 4:**
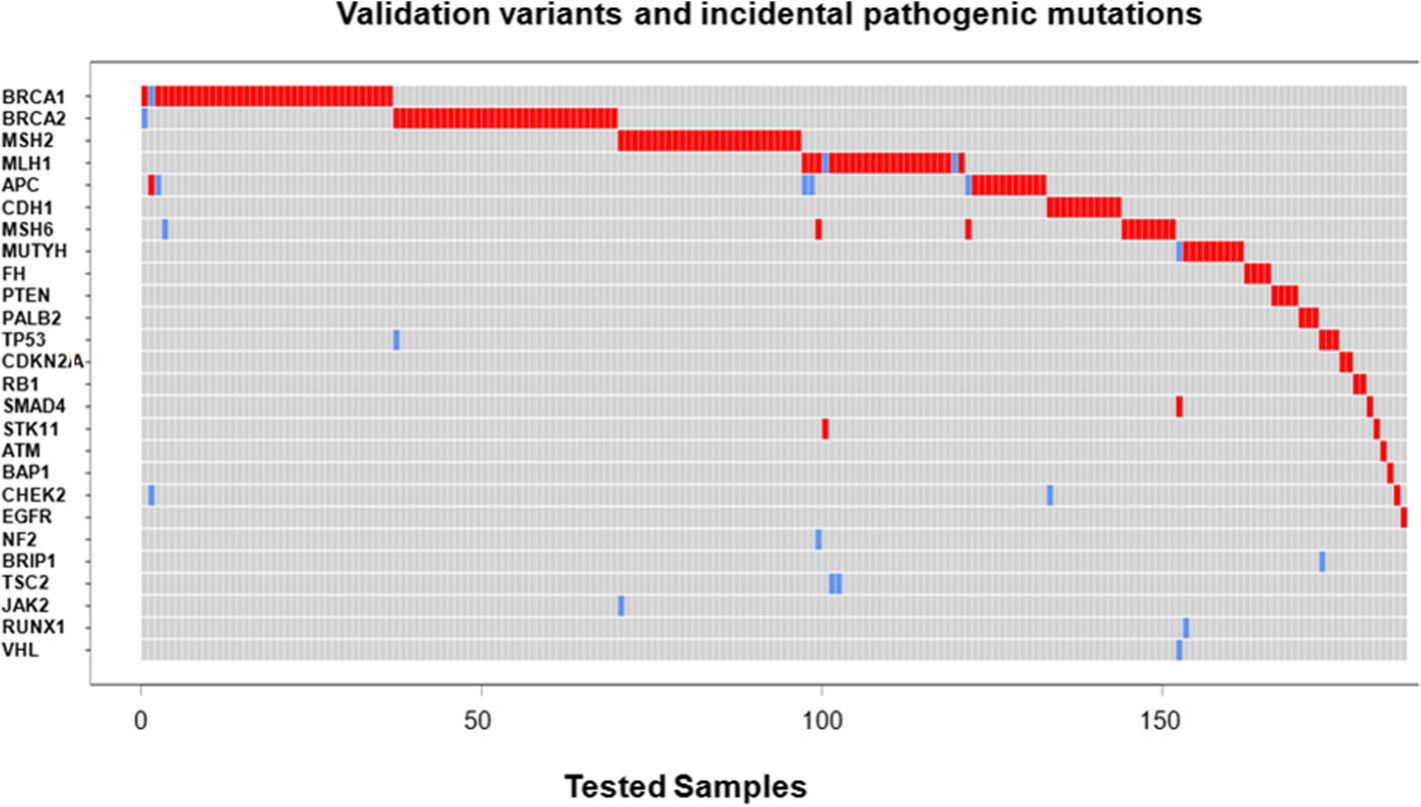
Distribution of expected variants vs. incidental pathogenic variants. Oncoprint shows the distribution of expected variants (*red*) vs. incidental pathogenic variants (*blue*) across 233 unique samples used for the validation of germline SNVs and Indels

Upon review of the pathogenicity estimates of all variant calls (known and incidental), we found 16 samples (7%) that presented with additional incidental pathogenic or likely pathogenic variants other than the expected germline variants they were known to possess: most of these showed 1 additional incidental pathogenic variant, however two samples showed 2 incidental pathogenic variants (Table 2). Figure 4 shows the distribution of known mutations vs. incidental pathogenic/likely pathogenic variants across cases with previously confirmed SNVs and indels. The mutations in the cases with the highest number of pathogenic variants occurred in genes associated with similar syndromes, or were known to be prevalent in specific ethnic groups. For example, PT23 was positive for frameshift deletion in *MLH1*, which predisposes for Lynch Syndrome and presumably was the test indication motivating genetic testing in the first place. However, this sample was also positive for another frameshift mutation in *NF2* c.1702_1703delAG (p.Arg568fs), which would not have been uncovered by a panel focused solely on genes predisposing for colorectal cancer and gastrointestinal malignancies. PT219 was known to harbor a frameshift deletion in *SMAD4*, but incidental findings in *MUTYH* (p.Tyr179Cys) and *VHL* (p.Glu52*) were identified. Since biallelic mutations in *MUTYH* cause *MUTYH*-Associated Polyposis (MAP) and the sample does not have another co-occuring *MUTYH* mutation, this suggests that PT219 is a carrier of *MUTYH* mutation. In addition, the incidental finding of a nonsense mutation in *VHL* is especially interesting, since it predisposes for Von Hippel-Lindau (VHL) syndrome in an autosomal dominant manner. Lastly, PT179 was known to be positive for *APC* p.Ile1307Lys, but MSK-IMPACT testing revealed additional pathogenic variants, i.e., *CHEK2* p.Ser428Phe and *BRCA1* c. 68_69delAG (i.e., 185delAG) mutations, all of which are known to be founder mutations in Ashkenazi Jews. The presence of these co-occurring mutations, many of which would not have been uncovered by single gene testing by Sanger Sequencing or a more limited NGS panel, may support a broader approach for cancer genetics predisposition testing.

**Table 2:** Sixteen samples with incidental pathogenic/likely pathogenic variants found, in addition to known variants (previously confirmed)

Patient	Expected variant (previously confirmed)	Incidental pathogenic variants found
PT179	APC p.I1307K (c.3920 T > A)	CHEK2 p.S428F (c.1283C > T), BRCA1 p.E23fs (c.68_69delAG)
PT219	SMAD4 p.L414fs (c.1242_1245delAGAC)	MUTYH p.Y179C (c.536A > G), VHL p.E52* (c.154G > T)
PT78	BRCA1 (c.301 + 1G > A)	BRCA2 p.S1982fs (c.5946delT)
PT58	BRCA1 Intragenic deletion exons 14 to 20	ATM p.V2497fs (c.7489_7490insTT)
PT57	BRCA2 p.D1898C (c.5692_5693delinsTG)	TP53 p.T123fs (c.368_369delCT)
PT151	CDH1 p.I363fs (c.1089_1090insACAGTCACTGACACCA)	CHEK2 p.T367fs (c.1100delC)
PT119	MLH1 Intragenic deletion exons 1-15	CHEK2 p.S428F (c.1283C > T)
PT187	MLH1 p.E331fs (c.992delA)	APC p.I1307K (c.3920 T > A)
PT23	MLH1 p.S388fs (c.1163_1164delCC)	NF2 p.R568fs (c.1702_1703delAG)
PT100	MLH1 p.Y548fs (c.1642_1648delTACCTTC)	TSC2 p.1684_1690del (c.5051_5068del)
PT141	MSH2 p.A636P (c.1906G > C)	JAK2 p.R761fs (c.2281_2282delAG)
PT212	MSH6 p.N1327fs (c.3980_3981insTCAG)	APC p.I1307K (c.3920 T > A)
PT145	MUTYH (c.892-2A > G)	RUNX1 p.K152fs (c.455dupA)
PT33	TP53 p.R342* (c.1024C > T)	BRIP1 p.N590fs (c.1770delC)
PT90	MLH1 p.A441T (c.1321G > A)	TSC2 p.1684_1690del (c.5051_5068del)
PT106	MLH1 p.T117R (c.350C > G)	APC p.I1307K (c.3920 T > A)

All detected mutations are heterozygous

## Discussion

Using an extensive set of 228 samples with known germline mutations/variants, we have performed a thorough analytic validation of the MSK-IMPACT panel as a targeted sequencing assay for detecting a variety of germline variants – SNVs, Indels, large deletions/duplications (CNVs) and structural variants (SVs). The technical reproducibility of the variant calls is high, as assessed by both within- and across-batch replicates. Unlike whole exome sequencing, MSK-IMPACT, being a targeted panel assay, is focused on a limited set of genes and its scope is hence limited to reporting mutations related to cancer predisposition and somatic tumorigenesis. This reduces the burden and potential issues arising from detecting incidental pathogenic variants in genes outside the scope of the requested test indication – for instance, out of the 76 genes of clinical interest in MSK-IMPACT, only 26 genes overlap the ACMG list of recommended genes for reporting incidental findings. Additionally, since the baits in MSK-IMPACT were deliberately designed to fully tile exon-intron boundaries, the increased depth of coverage permits an analysis of intronic regions immediately flanking the targeted exons.

Our analysis pipeline uses a number of recommended ‘best-practice’ algorithms for detecting variants (MuTect and GATK HaplotypeCaller for SNVs and Indels, DELLY for structural rearrangements) and we have even developed in-house and benchmarked a custom algorithm for detecting whole and partial-gene germline CNVs.

We found a number of incidental findings, i.e., cases with pathogenic variants not previously identified, distinct from the mutations for which these cases were known to harbor and specifically selected for validation. These cases constituted about 7.5% of the sequenced samples, suggesting that while uncommon, cases with multiple pathogenic variants may occur at a substantial frequency within a clinical setting. This may not be unexpected since testing is performed on an enriched population of patients with suspected hereditary cancer syndromes. While some cases were co-occurring founder mutations, several cases had co-occurring pathogenic mutations in genes predisposing towards different cancer syndromes. Traditional single gene sequencing approaches, or even more limited panel-based NGS assays, would not have uncovered these additional pathogenic mutations, reinforcing arguments for a broader approach for NGS testing of cancer predisposition genes. Increasing the scope of genetic testing, when taken to an extreme, may advocate for using whole genome or exome sequencing as a standard in clinical cancer genetics testing, however the burden of incidental findings in non-cancer related genes may be too high with such an approach. Using a broad based, targeted sequencing panel focused on cancer predisposition genes, such as MSK-IMPACT, may provide a middle ground, allowing for completeness of coverage in terms of cancer predisposition, while mitigating potential complications from detecting incidental findings in non-cancer related genes.

The importance of a matched normal in clinical tumor sequencing was underscored by a recent paper by Jones et al. [21], where the authors showed that lack of a matched normal could lead to increased reporting of germline private variants as somatic ‘false-positive’ mutations. In line with these findings, we routinely perform MSK-IMPACT as a matched tumor/normal sequencing test at our institution. Our efforts to validate MSK-IMPACT as a germline test will not only enable its use as a dedicated test in the clinical setting for diagnosis of cancer predisposition syndromes, but will also enable the concurrent, unambiguous identification of somatic mutations and inherited germline variation in the matched tumor/normal setting. When performed at scale, this presents a unique opportunity to simultaneously interrogate the spectrum of somatic and germline mutations in cancer, with a high accuracy, comprehensive, targeted sequencing platform.

## Conclusions

This study highlights the importance of the NGS-based gene panel testing approach in comprehensively identifying germline variants contributing to cancer predisposition. As MSK-IMPACT is also designed for use in paired tumor:normal sequencing, it enables simultaneous detection of somatic and germline alterations in that setting.

## Additional files

Additional file 1: Supplementary Methods and **Tables S1-S6**. (DOCX 93 kb)

Additional file 2: Figure S4. Allele frequency for non-reproducible false positive variants. Allele frequency (VF – x axis) is plotted against sequence coverage depth (DP – y axis) for non-reproducible false positive A) SNVs and B) indels, obtained from an analysis of replicates of thirteen reference blood normal samples. Dotted lines indicate filtering thresholds of DP=50X (horizontal) and VF=20% (Exonic variants) or 25% (Non-coding variants) (vertical). (TIF 123 kb)

Additional file 3: Figure S1. Sequence coverage for detected SNV and Indel variants. Sequence coverage (y-axis) is plotted against allele frequency (x-axis) for detected SNV and Indel variants: red = expected variants previously confirmed by independent methods, black = incidental variants. SNV and Indel variants located in exonic or non-coding regions are plotted separately. (TIF 157 kb)

Additional file 4: Figure S2. Examples of copy number plots indicating germline CNVs detected by MSK-IMPACT. A) intragenic deletion of BRCA2 exons 2 to 11, B) whole gene deletion of MSH2, C) intragenic duplication of BRCA2 exons 5 to 11. The y axis indicates log2 ratio of normalized coverage, comparing tested samples vs. reference diploid normals. The x axis depicts relative chromosomal positions of exonic (blue) and tiling (brown) regions. (TIF 182 kb)

Additional file 5: Figure S3. IGV screenshot illustrating an intragenic deletion in BRCA1. The deletion begins in the middle of exon 10 and extends into intron 18. Brown reads represent read pairs containing the mutation event. (TIF 223 kb)

## Abbreviations

CLIA: Clinical laboratory improvement amendments
CNVs: Copy number variants
HTC: HaplotypeCaller
Indels: Small insertions/deletions
MLPA: Multiplex ligation-dependent probe amplification
MSK-IMPACT: Integrated mutation profiling of actionable cancer targets
NGS: Next generation sequencing
SNVs: Single nucleotide variants
SVs: Structural variants
