# Supplementary material for: Comprehensive detection of germline variants by MSK-IMPACT, a clinical diagnostic platform for solid tumor molecular oncology and concurrent cancer predisposition testing
Source: BMC Med Genomics. 2017 May 19;10:33. doi: 10.1186/s12920-017-0271-4 (PMC5437632; doi:10.1186/s12920-017-0271-4)
Supplement: Supplementary file 1 — Supplementary Methods and Tables S1-S6. (DOCX 93 kb) [file 12920_2017_271_MOESM1_ESM.docx]

# SUPPLEMENTARY METHODS AND TABLES:

*Power analysis for establishing coverage thresholds for sensitivity*

We sought to determine the minimum level of coverage necessary to ensure sufficient statistical power for the detection of germline variants. Using a binomial model, we performed a power analysis to compute the expected sample size (i.e. number of reads) needed to detect heterozygous germline mutations (i.e. 50% allele frequency) for varying levels of power (0.8 to 0.99), assuming a fixed Type I error rate of 0.05 (**Supplementary Table 5**). The analysis showed that with 17X coverage, we would be able to avoid false negatives with 99% confidence – that is, if an exon was sequenced to at least 17X without mutations, there is a less than 1% chance that it actually contains a germline mutation that we did not sequence deeply enough to detect. Coverage in MSK-IMPACT routinely exceeds 17X in both exonic and flanking intronic regions, and from **Supplementary Table 5**, it may even be powered to detect somatic mosaic variants down to levels as low as 10 - 20%.

*Limits on coverage and allele frequency to reject assay false positives*

We sought to identify thresholds on parameters such as coverage, number of mutant reads and allele frequency, to flag variants as potential false positives (systematic assay and noise artifacts) and subsequently remove them. Systematic assay artifacts are highly reproducible across unrelated samples and can be identified based on their high call rates across a panel of unrelated reference normals. We considered variants with less than 1% prevalence in the general population (1000 Genomes, NHLBI ESP) that occurred repeatedly in our sequenced samples (> 5%) as systematic assay artifacts. To characterize random noise artifacts, we performed variant calling on replicates of thirteen blood samples from disease free individuals, and assessed the reproducibility of calls between replicates. Non-reproducible calls were attributed to noise, i.e. technical variability in the assay, and their respective coverage and allele frequency values are plotted in **Supplementary Figure 5**. We identified a total of 4696 exonic and 7986 flanking intronic SNVs, of which 96 (2%) and 500 (6%) were not reproducible between replicates. Similarly, a total of 128 exonic and 3081 flanking intronic indels were identified, of which 47 (36%) and 1003 (33%) were not reproducible. In reviewing the distribution of sequencing coverage and variant frequencies for these mutations, we observed that essentially all of the noise artifacts could be filtered out with a coverage depth threshold of 50X and variant frequency threshold of 20% for exonic variants, 25% for variants in flanking intronic regions. For exonic variants, these filtering criteria rejected all non-reproducible noise artifacts for both SNVs and Indels. For non-reproducible variants found in flanking intronic regions, these criteria resulted in a rejection rate of 99.2% (496/500) for SNVs and 99.5% (998/1003) for indels (**Supplementary Table 6**).

*Germline CNV analysis*

Coverage of targeted regions is computed using the GATK DepthOfCoverage tool, square-root transformed and adjusted for GC content using a Loess normalization procedure. Twenty blood normal samples from disease-free individuals were used as reference controls. These normal blood samples were anonymized. We run these samples against each other to make sure they do not have any alterations that pass our thresholds and affect our analysis of other samples. We are aware there are copy number variations in normal human population but the DNA samples we used as references seem to be free of alterations detectable by our pipeline. Normalized coverage values from germline samples are divided by their corresponding values in reference controls and log-transformed to yield log-ratios. Normalized values are segmented using Circular Binary Segmentation (CBS) and then grouped into clusters to identify diploid vs. non-diploid segments. Target regions from the segment cluster with mean log-ratio closest to 0 are used to parameterize a null distribution for estimating significance of copy number events. Whole and partial-gene CNVs are called by comparing the fold-changes and p-values of the non-diploid segments, using the diploid cluster segments as a reference. The following criteria is used to determine significance of gain or loss events: fold change > 1.3 (single copy gain) or < -1.7 (single copy loss), p < 0.05.

Large deletions/duplications can also be detected as structural variants, in cases where the aberration is caused by a genomic rearrangement and one end of the breakpoint is located in a genomic region captured by the panel bait set. DELLY version 0.3.3[[11](#_ENREF_11)] was used to detect structural variants in germline samples, using an unmatched reference normal as a control. DELLY requires both paired-read & split-read support to nominate rearrangement breakpoints; using a paired-sample calling procedure, each structural aberration detected in the analyzed sample is also evaluated in the comparator reference control. Variants co-occurring in the reference control are likely to be systematic sequencing/mapping artifacts and are filtered out as potential false positives. Rearrangements are also filtered for the following criteria before manual review: 5 paired or split reads, mapping quality ≥ 20, length > 500bp. All candidate structural aberrations were annotated using in-house tools, and manually reviewed using the Integrative Genomics Viewer (IGV) [21].

**Supplementary Table 1**: List of 76 genes of interest for germline cancer predisposition

| **Gene** | **Transcript ID** | **ACMG Incidental Findings list (n=26)** | **Diseases/Syndromes** |
| --- | --- | --- | --- |
| ALK | NM_004304 |  | Familial neuroblastoma |
| APC | NM_000038 | ACMG | Familial adenomatous polyposis |
| ATM | NM_000051 |  | Ataxia-telangiectasia; ATM-related cancer risk |
| BAP1 | NM_004656 |  | Mesothelioma, uveal melanoma |
| BARD1 | NM_000465 |  | Hereditary breast and ovarian cancer syndrome |
| BLM | NM_000057 |  | Bloom syndrome |
| BMPR1A | NM_004329 |  | Juvenile polyposis syndrome |
| BRCA1 | NM_007294 | ACMG | Hereditary breast and ovarian cancer syndrome |
| BRCA2 | NM_000059 | ACMG | Hereditary breast and ovarian cancer syndrome; Fanconi anemia |
| BRIP1 | NM_032043 |  | BRIP1-related cancer; Fanconi anemia |
| CDH1 | NM_004360 |  | Hereditary diffuse gastric cancer |
| CDK4 | NM_000075 |  | Familial cutaneous melanoma |
| CDKN2A | NM_000077 |  | Familial cutaneous melanoma |
| CHEK2 | NM_007194 |  | CHEK2-related cancer |
| DICER1 | NM_030621 |  | Pleuropulmonary blastoma |
| EGFR | NM_005228 |  | Familial lung cancer |
| EPCAM | NM_002354 |  | Lynch syndrome |
| FAM175A | NM_139076 |  | Hereditary breast cancer syndrome |
| FH | NM_000143 |  | Hereditary Leiomyomatosis and Renal Cell Cancer |
| FLCN | NM_144997 |  | Birt-Hogg-Dubé syndrome |
| GATA2 | NM_032638 |  | Familial MDS-AML |
| GREM1 | NM_013372 |  | Hereditary mixed polyposis syndrome (HMPS) |
| HRAS | NM_001130442 |  | Costello syndrome |
| JAK2 | NM_004972 |  | Familial thrombocytosis |
| KIT | NM_000222 |  | Hereditary Gastrointestinal stromal tumors (GISTs) |
| KRAS | NM_033360 |  | Noonan Syndrome |
| MAX | NM_002382 |  | Hereditary paraganglioma-pheochromocytoma (PGL/PCC) syndromes |
| MEN1 | NM_000244 | ACMG | Multiple endocrine neoplasia, type 1 |
| MET | NM_000245 |  | Hereditary papillary renal carcinoma |
| MITF | NM_000248 |  | Familial melanoma and renal cell carcinoma |
| MLH1 | NM_000249 | ACMG | Lynch syndrome |
| MRE11A | NM_005591 |  | Ataxia-telangiectasia-like disorder (recessive); breast cancer |
| MSH2 | NM_000251 | ACMG | Lynch syndrome |
| MSH6 | NM_000179 | ACMG | Lynch syndrome |
| MUTYH | NM_001128425 | ACMG | MUTYH-associated polyposis (MAP) |
| NBN | NM_002485 |  | Nijmegen breakage syndrome; NBN-related cancer risk |
| NF1 | NM_001042492 |  | Neurofibromatosis, type 1 |
| NF2 | NM_000268 | ACMG | Neurofibromatosis, type 2 |
| NRAS | NM_002524 |  | Autoimmune lymphoproliferative syndrome (ALPS) |
| PALB2 | NM_024675 |  | PALB2-related cancer; Fanconi anemia |
| PAX5 | NM_016734 |  | B cell precursor acute lymphoblastic leukemia (B-ALL) |
| PDGFRA | NM_006206 |  | Hereditary Gastrointestinal stromal tumors (GISTs) |
| PHOX2B | NM_003924 |  | Familial neuroblastoma; Congenital central hypoventilation syndrome (CCHS) |
| PMS2 | NM_000535 | ACMG | Lynch syndrome |
| POLE | NM_006231 |  | Colorectal cancer and Endometrial cancer |
| PTCH1 | NM_000264 |  | Nevoid basal cell carcinoma syndrome (NBCCS) |
| PTEN | NM_000314 | ACMG | PTEN hamartoma tumor syndrome |
| RAD50 | NM_005732 |  | Nijmegen breakage syndrome-like disorder |
| RAD51 | NM_002875 |  | Hereditary breast cancer |
| RAD51B | NM_133509 |  | Hereditary breast cancer |
| RAD51C | NM_058216 |  | RAD51C-related cancer; Fanconi anemia |
| RAD51D | NM_002878 |  | Hereditary ovarian cancer |
| RB1 | NM_000321 | ACMG | Retinoblastoma |
| RECQL4 | NM_004260 |  | Rothmund-Thomson syndrome (RTS) |
| RET | NM_020975 | ACMG | Multiple endocrine neoplasia, type 2 |
| RUNX1 | NM_001754 |  | Familial platelet disorder with predisposition to acute myelogenous leukaemia (FPD/AML) |
| SDHA | NM_004168 |  | Hereditary paraganglioma-pheochromocytoma (PGL/PCC) syndromes |
| SDHAF2 | NM_017841 | ACMG | Hereditary paraganglioma-pheochromocytoma (PGL/PCC) syndromes |
| SDHB | NM_003000 | ACMG | Hereditary paraganglioma-pheochromocytoma (PGL/PCC) syndromes |
| SDHC | NM_003001 | ACMG | Hereditary paraganglioma-pheochromocytoma (PGL/PCC) syndromes |
| SDHD | NM_003002 | ACMG | Hereditary paraganglioma-pheochromocytoma (PGL/PCC) syndromes |
| SMAD3 | NM_005902 | ACMG | Thoracic aortic aneurysms and aortic dissections (TAAD) |
| SMAD4 | NM_005359 |  | Juvenile polyposis syndrome |
| SMARCA4 | NM_003072 |  | Rhabdoid tumour predisposition syndrome type 2 |
| SMARCB1 | NM_003073 |  | Rhabdoid tumour predisposition syndrome type 1 |
| STK11 | NM_000455 | ACMG | Peutz-Jeghers syndrome |
| SUFU | NM_016169 |  | Medulloblastoma |
| TERT | NM_198253 |  | Familial pulmonary fibrosis (FPF); Dyskeratosis congenita (DC) |
| TGFBR1 | NM_004612 | ACMG | Thoracic aortic aneurysms and aortic dissections (TAAD) |
| TGFBR2 | NM_001024847 | ACMG | Thoracic aortic aneurysms and aortic dissections (TAAD) |
| TMEM127 | NM_001193304 |  | Familial pheochromocytoma syndrome |
| TP53 | NM_000546 | ACMG | Li-Fraumeni syndrome |
| TSC1 | NM_000368 | ACMG | Tuberous sclerosis complex (TSC) |
| TSC2 | NM_000548 | ACMG | Tuberous sclerosis complex (TSC) |
| VHL | NM_000551 | ACMG | Von Hippel-Lindau syndrome; Familial erythrocytosis, type 2 |
| WT1 | NM_024426 | ACMG | *WAGR (W*ilms tumor-*a*niridia-*g*enital anomalies-*r*etardation) syndrome, Denys-Drash syndrome (DDS), Frasier syndrome, and isolated Wilms tumor |

**Supplementary** **Table 2A**: Samples (189) with previously confirmed germline SNVs and indels validated by MSK-IMPACT.

| **Gene** | **Exon** | **cDNA Change** | **AA change** | **Variant Type** |
| --- | --- | --- | --- | --- |
| APC | 4 | c.221-2A>G |  | SNV |
| APC | 5 | c.505_508delATAG | p.I169fs | Indel |
| APC | 8 | c.776G>A | p.R259Q | SNV |
| APC | 16 | c.2738dupA | p.H913fs | Indel |
| APC | 16 | c.8291C>G | p.S2764C | SNV |
| APC | 16 | c.3920T>A | p.I1307K | SNV |
| APC | 16 | c.4824_4827delinsTAC | p.K1608fs | Indel |
| APC | 16 | c.6525A>G | p.T2175T | SNV |
| APC | 16 | c.3920T>A | p.I1307K | SNV |
| APC | 16 | c.6196A>G | p.R2066G | SNV |
| APC | 16 | c.3949G>C | p.E1317Q | SNV |
| APC | 16 | c.4611_4612delAG | p.T1537fs | Indel |
| APC | 16 | c.3386T>C | p.L1129S | SNV |
| ATM | 10 | c.1402_1403delAA | p.K468fs | Indel |
| BAP1 | 8 | c.639dupT | p.I214fs | Indel |
| BRCA1 | 2 | c.66dupA | p.E23fs | Indel |
| BRCA1 | 2 | c.53T>C | p.M18T | SNV |
| BRCA1 | 2 | c.68_69delAG | p.E23fs | Indel |
| BRCA1 | 2 | c.68_69delAG | p.E23fs | Indel |
| BRCA1 | 3 | c.117_118delTG | p.C39fs | Indel |
| BRCA1 | 3 | c.122A>G | p.H41R | SNV |
| BRCA1 | 4 | c.181T>G | p.C61G | SNV |
| BRCA1 | 6 | c.301+1G>A |  | SNV |
| BRCA1 | 7 | c.470_471delCT | p.S157fs | Indel |
| BRCA1 | 8 | c.548-9del |  | Indel |
| BRCA1 | 10 | c.1251_1252delinsA | p.N417fs | Indel |
| BRCA1 | 10 | c.3052_3053insTGAGA | p.N1018fs | Indel |
| BRCA1 | 10 | c.798_799delTT | p.V266fs | Indel |
| BRCA1 | 10 | c.3228_3229delAG | p.R1076fs | Indel |
| BRCA1 | 10 | c.3756_3759delGTCT | p.L1252fs | Indel |
| BRCA1 | 10 | c.1116G>A | p.W372X | SNV |
| BRCA1 | 10 | c.1961delA | p.K654fs | Indel |
| BRCA1 | 10 | c.1088delA | p.N363fs | Indel |
| BRCA1 | 10 | c.2722G>T | p.E908X | SNV |
| BRCA1 | 10 | c.2934T>G | p.Y978X | SNV |
| BRCA1 | 10 | c.3756_3759delGTCT | p.L1252fs | Indel |
| BRCA1 | 10 | c.2934T>G | p.Y978X | SNV |
| BRCA1 | 10 | c.3908dupT | p.L1303fs | Indel |
| BRCA1 | 10 | c.3700_3704delGTAAA | p.V1234fs | Indel |
| BRCA1 | 10 | c.1088delA | p.N363fs | Indel |
| BRCA1 | 12 | c.4327C>T | p.R1443X | SNV |
| BRCA1 | 13 | c.4484G>T | p.R1495M | SNV |
| BRCA1 | 14 | c.4574_4575delAA | p.Q1525fs | Indel |
| BRCA1 | 15 | c.4964_4982delCTGGCCTGACCCCAGAAGA | p.S1655fs | Indel |
| BRCA1 | 15 | c.4964_4982delCTGGCCTGACCCCAGAAGA | p.S1655fs | Indel |
| BRCA1 | 16 | c.5035delC | p.L1679X | Indel |
| BRCA1 | 17 | c.5096G>A | p.R1699Q | SNV |
| BRCA1 | 19 | c.5266dupC | p.Q1756fs | Indel |
| BRCA1 | 20 | c.5302T>G | p.C1768G | SNV |
| BRCA1 | 23 | c.5479_5480insGA | p.M1827fs | Indel |
| BRCA1 | 23 | c.5407-1G>C |  | SNV |
| BRCA2 | 3 | g.32893467G>A (c.316+5G>A) |  | SNV |
| BRCA2 | 4 | c.343A>G | p.K115E | SNV |
| BRCA2 | 5 | c.427dupC | p.S142fs | Indel |
| BRCA2 | 9 | c.710A>G | p.D237G | SNV |
| BRCA2 | 10 | c.1189_1190insTTAG | p.Q397fs | Indel |
| BRCA2 | 11 | c.4029_4033delAAATG | p.K1343fs | Indel |
| BRCA2 | 11 | c.6402_6406delTAACT | p.N2134fs | Indel |
| BRCA2 | 11 | c.5217_5223delTTTAAGT | p.Y1739fs | Indel |
| BRCA2 | 11 | c.4131_4132insTGAGGA | p.N1377delinsNX | Indel |
| BRCA2 | 11 | c.5645C>A | p.S1882X | SNV |
| BRCA2 | 11 | c.3922G>T | p.E1308X | SNV |
| BRCA2 | 11 | c.4944_4945delAA | p.A1648fs | Indel |
| BRCA2 | 11 | c.4165T>G | p.F1389V | SNV |
| BRCA2 | 11 | c.5574_5577delAATT | p.T1858fs | Indel |
| BRCA2 | 11 | c.5692_5693delinsTG | p.D1898C | Indel |
| BRCA2 | 11 | c.5569_5573delGAAAC | p.E1857fs | Indel |
| BRCA2 | 11 | c.5797_5800delAACC | p.N1933fs | Indel |
| BRCA2 | 11 | c.5067dupA | p.A1689fs | Indel |
| BRCA2 | 11 | c.6078dupA | p.T2026fs | Indel |
| BRCA2 | 11 | c.6491_6494delAGTT | p.Q2164fs | Indel |
| BRCA2 | 11 | c.6644_6647delACTC | p.Y2215fs | Indel |
| BRCA2 | 11 | c.5211_5214delTACT | p.D1737fs | Indel |
| BRCA2 | 11 | c.5211_5214delTACT | p.D1737fs | Indel |
| BRCA2 | 11 | c.2806_2809delAAAC | p.K936fs | Indel |
| BRCA2 | 12 | c.6853A>G | p.I2285V | SNV |
| BRCA2 | 14 | c.7057G>C | p.G2353R | SNV |
| BRCA2 | 14 | c.7097dupT | p.L2366fs | Indel |
| BRCA2 | 15 | c.7503_7506delACGC | p.Q2501fs | Indel |
| BRCA2 | 17 | c.7910_7914delCCTTT | p.A2637fs | Indel |
| BRCA2 | 18 | c.8009C>T | p.S2670L | SNV |
| BRCA2 | 23 | c.9076C>G | p.Q3026E | SNV |
| BRCA2 | 27 | c.9649-8T>C |  | SNV |
| BRCA2 | 27 | c.10121C>T | p.T3374I | SNV |
| CDH1 | 1 | c.3G>A | p.M1I | SNV |
| CDH1 | 3 | c.283C>T | p.Q95X | SNV |
| CDH1 | 5 | c.532-1G>A |  | SNV |
| CDH1 | 7 | c.1003C>T | p.R335X | SNV |
| CDH1 | 8 | c.1089_1090insACAGTCACTGACACCA | p.I363fs | Indel |
| CDH1 | 10 | c.1458_1459delTG | p.F486fs | Indel |
| CDH1 | 10 | c.1565+1G>A |  | SNV |
| CDH1 | 10 | c.1565+1G>A |  | SNV |
| CDH1 | 12 | c.1893dupA | p.T631fs | Indel |
| CDH1 | 14 | c.2287G>T | p.E763X | SNV |
| CDH1 | 15 | c.2323_2335delGGCCTGGACGCTC | p.G775fs | Indel |
| CDKN2A | 2 | c.334C>G | p.R112G | SNV |
| CDKN2A | 2 | c.334C>G | p.R112G | SNV |
| CHEK2 | 11 | c.1283C>T | p.S428F | SNV |
| EGFR | 20 | c.2369C>T | p.T790M | SNV |
| FH | 1 | c.132+5_+6delinsTG |  | SNV |
| FH | 7 | c.1083_1086delTGAA | p.N361fs | Indel |
| FH | 8 | c.1189G>A | p.G397R | SNV |
| FH | 8 | c.1138dupA | p.M380fs | Indel |
| MLH1 | 1 | c.22A>C | p.I8L | SNV |
| MLH1 | 1 | c.100_104delGAGAT | p.E34fs | Indel |
| MLH1 | 1 | c.71_75delTTATC | p.V24fs | Indel |
| MLH1 | 2 | c.154dupA | p.V51fs | Indel |
| MLH1 | 2 | c.154delA | p.K52fs | Indel |
| MLH1 | 4 | c.350C>G | p.T117R | SNV |
| MLH1 | 4 | c.350C>A | p.T117K | SNV |
| MLH1 | 7 | c.588+5G>T |  | SNV |
| MLH1 | 9 | c.739T>C | p.S247P | Indel |
| MLH1 | 10 | c.791_793delGATC | p.264_265del | Indel |
| MLH1 | 11 | c.954delC | p.H318fs | Indel |
| MLH1 | 11 | c.992delA | p.E331fs | Indel |
| MLH1 | 11 | c.954delC | p.H318fs | Indel |
| MLH1 | 12 | c.1321G>A | p.A441T | SNV |
| MLH1 | 12 | c.1367delC | p.S456X | Indel |
| MLH1 | 14 | c.1642_1648delTACCTTC | p.Y548fs | SNV |
| MLH1 | 16 | c.1855_1856insCTGA | p.A619fs | Indel |
| MLH1 | 16 | c.1845_1847delGAA | p.615_616del | Indel |
| MLH1 | 16 | c.1769_1772delTAGA | p.L590fs | Indel |
| MLH1 | 19 | c.2251_2252delAA | p.K751fs | Indel |
| MLH1 | 19 | c.2251_2252delAA | p.K751fs | Indel |
| MLH1 | 19 | c.2152C>T | p.H718Y | SNV |
| MLH1/MSH6 | 12 | c.1163_1164delCC/c.4065_4066insTTGA | p.S388fs/p.T1355fs | Indel |
| MSH2 | 2 | c.212-1G>A |  | SNV |
| MSH2 | 3 | c.484G>A | p.G162R | SNV |
| MSH2 | 5 | c.942+3A>T |  | SNV |
| MSH2 | 6 | c.943-1G>T |  | SNV |
| MSH2 | 6 | c.1032_1033insTTA | p.Q344_W345insL | Indel |
| MSH2 | 6 | c.970_971delCA | p.Q324fs | Indel |
| MSH2 | 7 | c.1229_1230insTATAAAT | p.G410fs | Indel |
| MSH2 | 7 | c.1147C>T | p.R383X | SNV |
| MSH2 | 7 | c.1164_1165delinsGT | p.388_389delinsKX | SNV |
| MSH2 | 7 | c.1216C>T | p.R406X | SNV |
| MSH2 | 8 | c.1278-14C>G |  | SNV |
| MSH2 | 10 | c.1566C>A | p.Y522X | SNV |
| MSH2 | 10 | c.1571G>A | p.R524H | SNV |
| MSH2 | 12 | c.1760_1781delACAGGCTATGTAGAACCAATGCAGAC | p.G587fs | Indel |
| MSH2 | 12 | c.1906G>C | p.A636P | SNV |
| MSH2 | 12 | c.1906G>C | p.A636P | SNV |
| MSH2 | 12 | c.1777C>T | p.Q593X | SNV |
| MSH2 | 12 | c.1847C>G | p.P616R | SNV |
| MSH2 | 12 | c.1906G>C | p.A636P | SNV |
| MSH2 | 12 | c.1906G>C | p.A636P | SNV |
| MSH2 | 12 | c.1906G>C | p.A636P | SNV |
| MSH2 | 12 | c.1906G>C | p.A636P | SNV |
| MSH2 | 12 | c.1906G>C | p.A636P | SNV |
| MSH2 | 12 | c.1906G>C | p.A636P | SNV |
| MSH2 | 12 | c.1906G>C | p.A636P | SNV |
| MSH2 | 12 | c.1906G>C | p.A636P | SNV |
| MSH2 | 12 | c.2003_2005delCTGGTAAAAAAC | p.668_669del | Indel |
| MSH2 | 13 | c.2168dupC | p.S723fs | Indel |
| MSH6 | 4 | c.3139delT | p.W1047fs | Indel |
| MSH6 | 4 | c.1312dupA | p.H437fs | Indel |
| MSH6 | 4 | c.1458_1459delTG | p.T486fs | Indel |
| MSH6 | 5 | c.3254dupC | p.T1085fs | Indel |
| MSH6 | 6 | c.3482_3484delCTG | p.1161_1162del | Indel |
| MSH6 | 8 | c.3722_3724delGTC | p.1241_1242del | Indel |
| MSH6 | 9 | c.3802-4insAAGGCATGCATGGTAGAAAATG |  | Indel |
| MSH6 | 9 | c.3846_3847insATTA | p.T1282fs | Indel |
| MSH6 | 9 | c.3980_3981insTCAG | p.N1327fs | Indel |
| MUTYH | 2 | c.53C>T | p.P18L | SNV |
| MUTYH | 7 | c.536A>G | p.Y179C | SNV |
| MUTYH | 7 | c.536A>G | p.Y179C | SNV |
| MUTYH | 10 | c.877_878insCTGTGGAGAGCCTGTGCC | p.R293_A294insPVESLC | Indel |
| MUTYH | 10 | c.925C>T | p.R309C | SNV |
| MUTYH | 12 | c.934-2A>G |  | SNV |
| MUTYH | 13 | c.1258C>A | p.L420M | SNV |
| MUTYH | 14 | c.1437_1439delGGA | p.479_480del | Indel |
| MUTYH | 15 | c.1518+4A>G |  | SNV |
| MUTYH | 16 | c.1601G>A | p.R534Q | SNV |
| PALB2 | 4 | c.925A>G | p.I309V | SNV |
| PALB2 | 7 | c.2590C>T | p.P864S | SNV |
| PALB2 | 9 | c.2858A>G | p.D953G | SNV |
| PTEN | 1 | c.43A>G | p.R15G | SNV |
| PTEN | 3 | c.165-20_c.165-24del |  | Indel |
| PTEN | 5 | c.389G>C | p.R130P | SNV |
| PTEN | 5 | c.389G>A | p.R130Q | SNV |
| RB1 | 12 | c.1215+1G>A |  | SNV |
| RB1 | 22 | c.2236delG | p.E746fs | Indel |
| SMAD4 | 10 | c.1242_1245delAGAC | p.L414fs | Indel |
| SMAD4 | 12 | c.1507_1508insATCC | p.M503fs | Indel |
| STK11 | 1 | c.179dupA | p.Y60_G61delinsX | Indel |
| STK11 | 5 | c.598-9C>G |  | SNV |
| TP53 | 2 | c.75+14T>C |  | SNV |
| TP53 | 3 | c.96+37G>A |  | SNV |
| TP53 | 10 | c.1024C>T | p.R342X | SNV |

**Supplementary** **Table 2B**: Samples (44) with previously confirmed germline CNVs validated by MSK-IMPACT.

| **Gene** | **Gain or loss** | **Exons affected** |
| --- | --- | --- |
| APC | Loss | All |
| ATM | Loss | 2,3,4,5,6,7,8,9,10,11 |
| BMPR1A | Loss | 5 |
| BRCA1 | Loss | 14,15,16,17,18,19,20 |
| BRCA1 | Gain | 12,13 |
| BRCA1 | Loss | 21,22,23,24 |
| BRCA1 | Loss | 2 |
| BRCA1 | Loss | 14,15,16,18,19,20 |
| BRCA1 | Loss | 21,22,23,24 |
| BRCA1 | Loss | 11,12,13,14,15,16,17,18 |
| BRCA1 | Gain | 3 |
| BRCA1 | Gain | 3 |
| BRCA1 | Loss | 19 |
| BRCA1 | Loss | 17,18 |
| BRCA1 | Loss | 19 |
| BRCA1 | Loss | 10,11,12,13,14 |
| BRCA2 | Gain | 5,6,7,8,9,10,11 |
| BRCA2 | Loss | 2,3,4,5,6,7,8,9,10,11 |
| BRCA2 | Loss | 2 |
| BRCA2 | Loss | 2 |
| BRCA2 | Loss | 14,15,16 |
| BRCA2 | Loss | 14,15,16,17,18 |
| CDH1 | Loss | All |
| EPCAM | Loss | All |
| FH | Loss | All |
| MLH1 | Loss | 3 |
| MLH1 | Loss | 14 |
| MLH1 | Loss | 1,2,3,4,5,6,7,8,9,10,11,12,13,14,15 |
| MLH1 | Loss | 14 |
| MLH1 | Gain | 16,17,18,19 |
| MSH2 | Gain | 1,2,3,4,5,6 |
| MSH2 | Loss | All |
| MSH2 | Loss | 8,9,10,11,12,13,14,15,16 |
| MSH2 | Loss | 1,2,3,4,5,6,7,8 |
| MSH2 | Loss | 1,2,3,4,5,6 |
| MSH2 | Loss | 3,4 |
| MSH2 | Loss | 3 |
| MSH2 | Loss | 3,4 |
| MSH2 | Loss | 2 |
| MSH2 | Loss | 7,8,9,10,11,12,13,14,15,16 |
| MSH2 | Loss | 8 |
| MSH2 | Loss | 9,10 |
| MSH2 | Gain | 11,12,13,14,15,16 |
| PALB2 | Gain | 13 |

**Supplementary Table 3:** Reproducibility of variant calls comparing intra- and inter-run replicates

|  | | | | **Number of Variants Reported** | | **Known Variant Statistics** | | |
| --- | --- | --- | --- | --- | --- | --- | --- | --- |
| **SNV and Indel samples** | **Barcode** | **Run** | **Sample Coverage (X)** | **Exonic**  **Variants** | **Noncoding Variants** | **Coverage (X)** | **Variant Frequency** | **Normalized Coverage** |
| *MSH2* exon 12  p.A636P | bc13 | 1 | 421 | 95 | 117 | 839 | 0.457 | 1.24 |
|  | bc14 | 1 | 909 | 95 | 117 | 752 | 0.483 | 1.25 |
|  | bc15 | 1 | 677 | 95 | 116 | 928 | 0.486 | 1.30 |
|  | bc02 | 2 | 601 | 95 | 116 | 448 | 0.502 | 1.06 |
|  | bc39 | 3 | 712 | 95 | 117 | 1107 | 0.527 | 1.22 |
| *BRCA1* exon 2  p.E23fs | bc07 | 1 | 698 | 96 | 115 | 783 | 0.489 | 1.25 |
|  | bc08 | 1 | 722 | 96 | 116 | 848 | 0.463 | 1.24 |
|  | bc09 | 1 | 624 | 96 | 114 | 688 | 0.464 | 1.17 |
|  | bc06 | 2 | 685 | 96 | 115 | 717 | 0.485 | 1.03 |
|  | bc27 | 3 | 586 | 96 | 116 | 897 | 0.502 | 1.24 |
| *BRCA1* exon 19 p.Q1756fs | bc19 | 1 | 1028 | 99 | 111 | 1206 | 0.476 | 2.10 |
|  | bc20 | 1 | 732 | 99 | 111 | 1340 | 0.465 | 2.15 |
|  | bc39 | 1 | 575 | 99 | 111 | 1310 | 0.470 | 2.06 |
|  | bc12 | 2 | 623 | 99 | 112 | 1907 | 0.449 | 1.86 |
|  | bc28 | 3 | 637 | 99 | 111 | 1576 | 0.489 | 2.15 |
| *BRCA2* exon 11 p.D1737fs | bc40 | 1 | 588 | 92 | 116 | 626 | 0.500 | 0.84 |
|  | bc41 | 1 | 794 | 92 | 116 | 713 | 0.442 | 0.88 |
|  | bc42 | 1 | 747 | 92 | 115 | 749 | 0.427 | 0.89 |
|  | bc29 | 2 | 813 | 92 | 115 | 428 | 0.488 | 0.73 |
|  | bc40 | 3 | 846 | 92 | 115 | 609 | 0.478 | 0.77 |
| **CNV samples**  **(large deletions only)** | **Barcode** | **Run** | **Sample Coverage (X)** | **Exonic**  **Variants** | **Noncoding Variants** | **Affected exons** | | **Fold change** |
| *BRCA1* large deletion | bc43 | 1 | 651 | 85 | 107 | 20,21,22,23 | | -2.00 |
|  | bc44 | 1 | 805 | 85 | 106 | 20,21,22,23 | | -2.02 |
|  | bc45 | 1 | 759 | 85 | 107 | 20,21,22,23 | | -2.07 |
|  | bc23 | 2 | 625 | 85 | 107 | 20,21,22,23 | | -1.99 |
|  | bc19 | 3 | 693 | 85 | 106 | 20,21,22,23 | | -2.01 |
| *MSH2* large deletion | bc10 | 1 | 431 | 97 | 123 | 1,2,3,4,5,6 | | -2.00 |
|  | bc11 | 1 | 975 | 97 | 123 | 1,2,3,4,5,6 | | -2.06 |
|  | bc12 | 1 | 802 | 97 | 122 | 1,2,3,4,5,6 | | -2.01 |
|  | bc34 | 2 | 803 | 97 | 120 | 1,2,3,4,5,6 | | -2.01 |
|  | bc20 | 3 | 727 | 97 | 122 | 1,2,3,4,5,6 | | -1.94 |

**Supplementary Table 4**: Comparison of intronic variants discrepant between replicates tested for reproducibility.

|  | Sample | Chrom | Start | Ref | Alt | VariantClass | Gene | Coverage (X) | Allele Freq |
| --- | --- | --- | --- | --- | --- | --- | --- | --- | --- |
| MSH2 exon 12  p.A636P | PT89 | 1 | 161293485 | T | TTA | intronic | SDHC | 327 | 0.33 |
|  | PT89-2 | 1 | 161293485 | T | TTA | intronic | SDHC | 593 | 0.34 |
|  | PT89-3A | 1 | 161293485 | T | TTA | intronic | SDHC | 473 | 0.35 |
|  | PT89-3B | 1 | 161293485 | T | TTA | intronic | SDHC | 488 | 0.26 |
|  | PT89-3C | 1 | 161293485 | T | TTA | intronic | SDHC | 479 | **0.24** |
|  | PT89 | 17 | 29541437 | T | C | intronic | NF1 | **39** | 1 |
|  | PT89-2 | 17 | 29541437 | T | C | intronic | NF1 | 71 | 1 |
|  | PT89-3A | 17 | 29541437 | T | C | intronic | NF1 | 56 | 1 |
|  | PT89-3B | 17 | 29541437 | T | C | intronic | NF1 | 50 | 1 |
|  | PT89-3C | 17 | 29541437 | T | C | intronic | NF1 | 55 | 1 |
| BRCA1 exon 2  p.E23fs | PT44 | 17 | 29541437 | T | C | intronic | NF1 | **36** | 1 |
|  | PT44-2 | 17 | 29541437 | T | C | intronic | NF1 | 60 | 1 |
|  | PT44-3A | 17 | 29541437 | T | C | intronic | NF1 | 53 | 0.98 |
|  | PT44-3B | 17 | 29541437 | T | C | intronic | NF1 | 51 | 1 |
|  | PT44-3C | 17 | 29541437 | T | C | intronic | NF1 | **37** | 1 |
|  | PT44 | 21 | 36164405 | G | T | UTR3 | RUNX1 | 105 | 0.59 |
|  | PT44-2 | 21 | 36164405 | G | T | UTR3 | RUNX1 | 85 | 0.55 |
|  | PT44-3A | 21 | 36164405 | G | T | UTR3 | RUNX1 | **48** | 0.68 |
|  | PT44-3B | 21 | 36164405 | G | T | UTR3 | RUNX1 | 61 | 0.50 |
|  | PT44-3C | 21 | 36164405 | G | T | UTR3 | RUNX1 | **43** | 0.58 |
| BRCA1 exon 19 p.Q1756fs | PT96 | 13 | 48954159 | C | CT | intronic | RB1 | 151 | 0.24 |
|  | PT96-2 | 13 | 48954159 | C | CT | intronic | RB1 | 130 | 0.27 |
|  | PT96-3A | 13 | 48954159 | C | CT | intronic | RB1 | 98 | 0.35 |
|  | PT96-3B | 13 | 48954159 | C | CT | intronic | RB1 | 100 | 0.25 |
|  | PT96-3C | 13 | 48954159 | C | CT | intronic | RB1 | 134 | 0.34 |
|  | PT96 | 17 | 29541437 | T | C | intronic | NF1 | 50 | 1 |
|  | PT96-2 | 17 | 29541437 | T | C | intronic | NF1 | **25** | 1 |
|  | PT96-3A | 17 | 29541437 | T | C | intronic | NF1 | **32** | 1 |
|  | PT96-3B | 17 | 29541437 | T | C | intronic | NF1 | **28** | 1 |
|  | PT96-3C | 17 | 29541437 | T | C | intronic | NF1 | **38** | 1 |
|  | PT96 | 3 | 70014447 | T | C | UTR3 | MITF | 56 | 0.57 |
|  | PT96-2 | 3 | 70014447 | T | C | UTR3 | MITF | **38** | 0.52 |
|  | PT96-3A | 3 | 70014447 | T | C | UTR3 | MITF | **32** | 0.37 |
|  | PT96-3B | 3 | 70014447 | T | C | UTR3 | MITF | **36** | 0.44 |
|  | PT96-3C | 3 | 70014447 | T | C | UTR3 | MITF | **31** | 0.29 |
| BRCA2 exon 11 p.D1737fs | PT87 | 17 | 29541437 | T | C | intronic | NF1 | **39** | 0.58 |
|  | PT87-2 | 17 | 29541437 | T | C | intronic | NF1 | **46** | 0.54 |
|  | PT87-3A | 17 | 29541437 | T | C | intronic | NF1 | 65 | 0.49 |
|  | PT87-3B | 17 | 29541437 | T | C | intronic | NF1 | 61 | 0.45 |
|  | PT87-3C | 17 | 29541437 | T | C | intronic | NF1 | **49** | 0.53 |
| BRCA1 large deletion | PT75 | 1 | 161293485 | T | TTA | intronic | SDHC | 512 | 0.34 |
|  | PT75-2 | 1 | 161293485 | T | TTA | intronic | SDHC | 550 | **0.21** |
|  | PT75-3A | 1 | 161293485 | T | TTA | intronic | SDHC | 497 | 0.31 |
|  | PT75-3B | 1 | 161293485 | T | TTA | intronic | SDHC | 393 | 0.30 |
|  | PT75-3C | 1 | 161293485 | T | TTA | intronic | SDHC | 483 | 0.27 |
|  | PT75 | 17 | 29541437 | T | C | intronic | NF1 | 50 | 1 |
|  | PT75-2 | 17 | 29541437 | T | C | intronic | NF1 | 60 | 1 |
|  | PT75-3A | 17 | 29541437 | T | C | intronic | NF1 | 74 | 0.98 |
|  | PT75-3B | 17 | 29541437 | T | C | intronic | NF1 | **46** | 1 |
|  | PT75-3C | 17 | 29541437 | T | C | intronic | NF1 | 60 | 1 |
| MSH2 large deletion | PT83 | 1 | 17354373 | G | GGAA | intronic | SDHB | 265 | 0.21 |
|  | PT83-2 | 1 | 17354373 | G | GGAA | intronic | SDHB | 658 | 0.24 |
|  | PT83-3A | 1 | 17354373 | G | GGAA | intronic | SDHB | 527 | 0.26 |
|  | PT83-3B | 1 | 17354373 | G | GGAA | intronic | SDHB | 522 | 0.25 |
|  | PT83-3C | 1 | 17354373 | G | GGAA | intronic | SDHB | 464 | 0.23 |
|  | PT83 | 14 | 68331675 | A | T | intronic | RAD51B | **37** | 1 |
|  | PT83-2 | 14 | 68331675 | A | T | intronic | RAD51B | 108 | 1 |
|  | PT83-3A | 14 | 68331675 | A | T | intronic | RAD51B | 97 | 1 |
|  | PT83-3B | 14 | 68331675 | A | T | intronic | RAD51B | 108 | 1 |
|  | PT83-3C | 14 | 68331675 | A | T | intronic | RAD51B | 76 | 1 |
|  | PT83 | 17 | 29541437 | T | C | intronic | NF1 | **25** | 0.72 |
|  | PT83-2 | 17 | 29541437 | T | C | intronic | NF1 | 58 | 0.34 |
|  | PT83-3A | 17 | 29541437 | T | C | intronic | NF1 | 74 | 0.52 |
|  | PT83-3B | 17 | 29541437 | T | C | intronic | NF1 | 67 | 0.47 |
|  | PT83-3C | 17 | 29541437 | T | C | intronic | NF1 | 61 | 0.57 |

**Supplementary Table 5**: Coverage (DP) required to detect a variant with variant frequency (VF) for a given level of power and specified Type I error rate (alpha=0.05). Minimum coverage needed to detect a heterozygous germline mutation is highlighted in bold.

|  | DP needed to attain specified power (alpha = 0.05) | | | | |
| --- | --- | --- | --- | --- | --- |
| VF | Power=0.8 | Power=0.9 | Power=0.95 | Power=0.98 | Power=0.99 |
| 0.1 | 60 | 73 | 85 | 100 | 110 |
| 0.2 | 26 | 31 | 36 | 42 | 46 |
| 0.3 | 16 | 20 | 23 | 26 | 29 |
| 0.4 | 12 | 14 | 16 | 19 | 21 |
| **0.5** | **9** | **11** | **13** | **15** | **17** |
| 0.6 | 7 | 9 | 10 | 12 | 14 |

**Supplementary Table 6**: Number of false positive events detected pre- and post-filtering for coverage depth (DP) and allele frequency (VF).

|  | Exonic Variants | | Flanking intronic sequence (50bp) | |
| --- | --- | --- | --- | --- |
| Filter criteria | DP ≥ 50X, VF ≥ 20% | | DP ≥ 50X, VF ≥ 25% | |
|  | SNVs | Indels | SNVs | Indels |
| Pre-filter | 96 | 47 | 500 | 1003 |
| Post-filter | 0 | 0 | 4 | 5 |
| Rejection Rate | 1.00 | 1.00 | 0.992 | 0.995 |
