# Supplementary figures and images for: Comprehensive detection of germline variants by MSK-IMPACT, a clinical diagnostic platform for solid tumor molecular oncology and concurrent cancer predisposition testing
Source: BMC Med Genomics. 2017 May 19;10:33. doi: 10.1186/s12920-017-0271-4 (PMC5437632; doi:10.1186/s12920-017-0271-4)

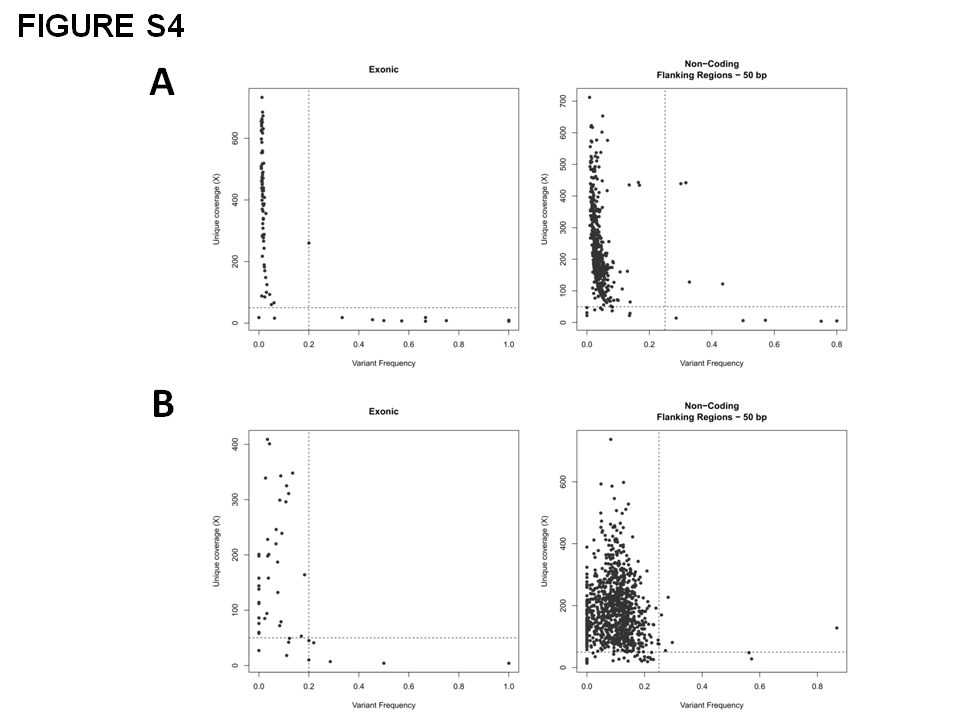

Supplement: Supplementary file 2 — Allele frequency for non-reproducible false positive variants. Allele frequency (VF – x axis) is plotted against sequence coverage depth (DP – y axis) for non-reproducible false positive A) SNVs and B) indels, obtained from an analysis of replicates of thirteen reference blood normal samples. Dotted lines indicate filtering thresholds of DP=50X (horizontal) and VF=20% (Exonic variants) or 25% (Non-coding variants) (vertical). (TIF 123 kb) [file 12920_2017_271_MOESM2_ESM.tif]

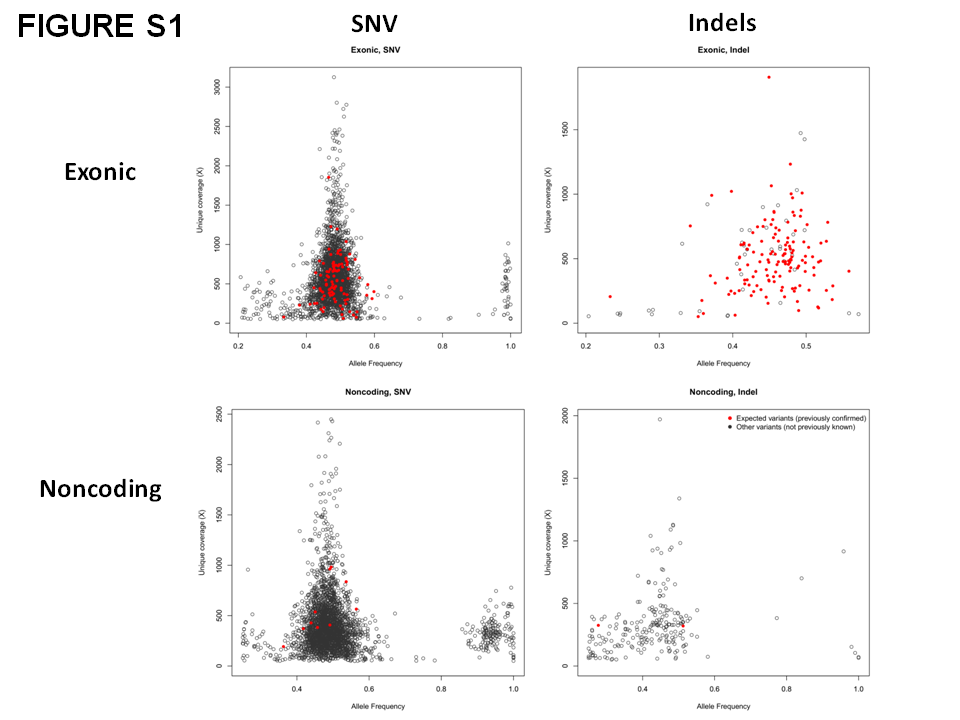

Supplement: Supplementary file 3 — Sequence coverage for detected SNV and Indel variants. Sequence coverage (y-axis) is plotted against allele frequency (x-axis) for detected SNV and Indel variants: red = expected variants previously confirmed by independent methods, black = incidental variants. SNV and Indel variants located in exonic or non-coding regions are plotted separately. (TIF 157 kb) [file 12920_2017_271_MOESM3_ESM.tif]

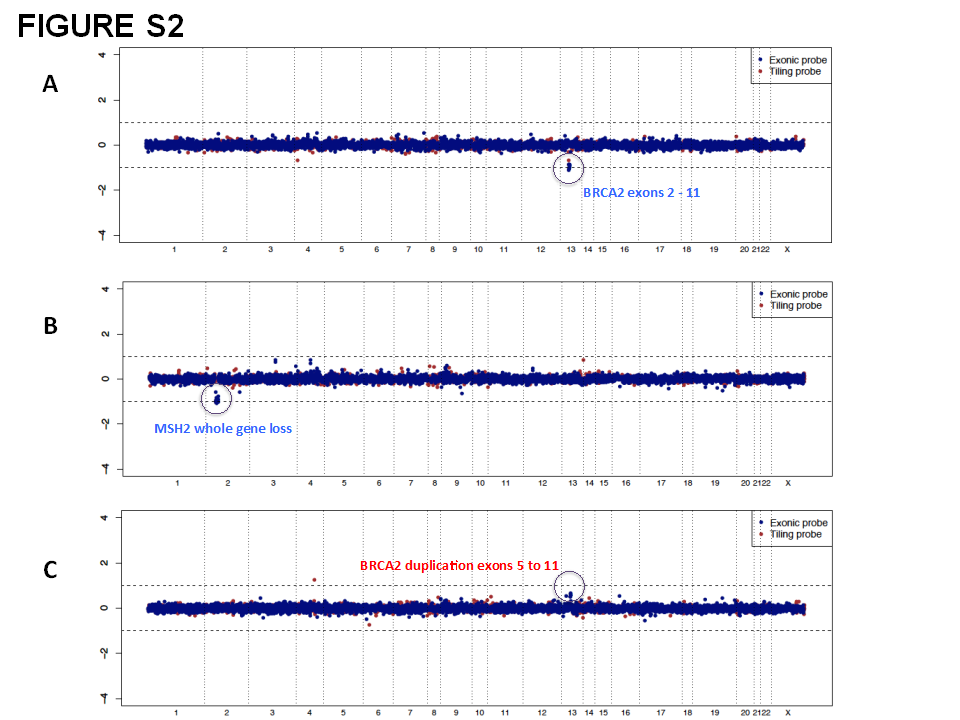

Supplement: Supplementary file 4 — Examples of copy number plots indicating germline CNVs detected by MSK-IMPACT. A) intragenic deletion of BRCA2 exons 2 to 11, B) whole gene deletion of MSH2, C) intragenic duplication of BRCA2 exons 5 to 11. The y axis indicates log2 ratio of normalized coverage, comparing tested samples vs. reference diploid normals. The x axis depicts relative chromosomal positions of exonic (blue) and tiling (brown) regions. (TIF 182 kb) [file 12920_2017_271_MOESM4_ESM.tif]

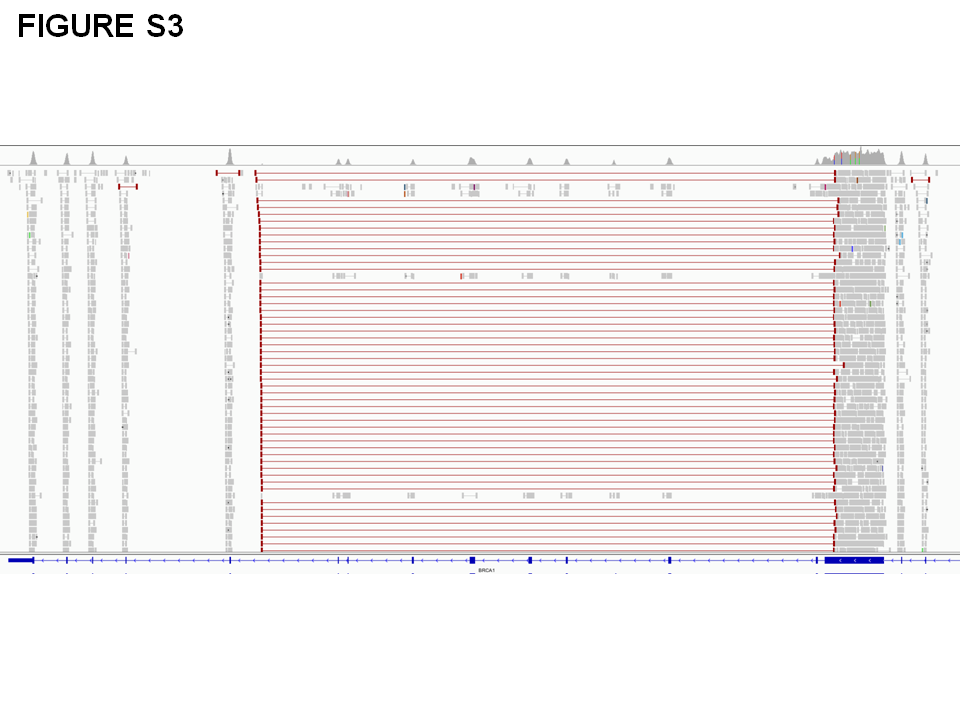

Supplement: Supplementary file 5 — IGV screenshot illustrating an intragenic deletion in BRCA1. The deletion begins in the middle of exon 10 and extends into intron 18. Brown reads represent read pairs containing the mutation event. (TIF 223 kb) [file 12920_2017_271_MOESM5_ESM.tif]
